# Classification of recovery states in U15, U17, and U19 sub-elite football players: a machine learning approach

**DOI:** 10.3389/fpsyg.2024.1447968

**Published:** 2024-10-29

**Authors:** José E. Teixeira, Samuel Encarnação, Luís Branquinho, Ricardo Ferraz, Daniel L. Portella, Diogo Monteiro, Ryland Morgans, Tiago M. Barbosa, António M. Monteiro, Pedro Forte

**Affiliations:** ^1^Department of Sports Sciences, Polytechnic of Guarda, Guarda, Portugal; ^2^Department of Sports Sciences, Polytechnic of Cávado and Ave, Guimarães, Portugal; ^3^SPRINT—Sport Physical Activity and Health Research & Inovation Center, Guarda, Portugal; ^4^Research Center in Sports, Health and Human Development, Covilhã, Portugal; ^5^LiveWell—Research Centre for Active Living and Wellbeing, Polytechnic Institute of Bragança, Bragança, Portugal; ^6^CI-ISCE, ISCE Douro, Penafiel, Portugal; ^7^Department of Sports Sciences, Universidad Autónoma de Madrid (UAM), Madrid, Spain; ^8^Department of Sports Sciences, Polytechnic Institute of Bragança, Bragança, Portugal; ^9^Biosciences Higher School of Elvas, Polytechnic Institute of Portalegre, Portalegre, Portugal; ^10^Life Quality Research Center (LQRC-CIEQV), Santarém, Portugal; ^11^Department of Sports Sciences, University of Beira Interior, Covilhã, Portugal; ^12^Group of Study and Research in Physical Exercise Science, University of São Caetano do Sul, São Caetano do Sul, Brazil; ^13^Master’s Programme in Innovation in Higher Education in Health, University of São Caetano do Sul, São Caetano do Sul, Brazil; ^14^ESECS-Polytechnic of Leiria, Leiria, Portugal; ^15^School of Sport and Health Sciences, Cardiff Metropolitan University, Cardiff, United Kingdom; ^16^Department of Sports Sciences, Higher Institute of Educational Sciences of the Douro, Penafiel, Portugal

**Keywords:** youth soccer, recovery, GPS, perceived exertion, AI

## Abstract

**Introduction:**

A promising approach to optimizing recovery in youth football has been the use of machine learning (ML) models to predict recovery states and prevent mental fatigue. This research investigates the application of ML models in classifying male young football players aged under (U)15, U17, and U19 according to their recovery state. Weekly training load data were systematically monitored across three age groups throughout the initial month of the 2019–2020 competitive season, covering 18 training sessions and 120 observation instances. Outfield players were tracked using portable 18-Hz global positioning system (GPS) devices, while heart rate (HR) was measured using 1 Hz telemetry HR bands. The rating of perceived exertion (RPE 6–20) and total quality recovery (TQR 6–20) scores were employed to evaluate perceived exertion, internal training load, and recovery state, respectively. Data preprocessing involved handling missing values, normalization, and feature selection using correlation coefficients and a random forest (RF) classifier. Five ML algorithms [K-nearest neighbors (KNN), extreme gradient boosting (XGBoost), support vector machine (SVM), RF, and decision tree (DT)] were assessed for classification performance. The K-fold method was employed to cross-validate the ML outputs.

**Results:**

A high accuracy for this ML classification model (73–100%) was verified. The feature selection highlighted critical variables, and we implemented the ML algorithms considering a panel of 9 variables (U15, U19, body mass, accelerations, decelerations, training weeks, sprint distance, and RPE). These features were included according to their percentage of importance (3–18%). The results were cross-validated with good accuracy across 5-fold (79%).

**Conclusion:**

The five ML models, in combination with weekly data, demonstrated the efficacy of wearable device-collected features as an efficient combination in predicting football players’ recovery states.

## 1 Introduction

Classifying recovery states in young football players who are still developing physically and mentally is crucial to ensure a high performance, reduce the injury risk, and enhance a better fatigue management (Rico-González et al., 2022b; Kellmann et al., 2018). Recovery management for under (U)15, U17, and U19 male football players must consider various physiological, psychological, and external factors that influence the effectiveness of rest and recuperation periods (Teixeira et al., 2022a; Teixeira et al., 2022b). Proper assessment and monitoring of recovery states can yield vital information about players’ readiness and overall health, thereby guiding coaches in tailoring training loads and recovery protocols more effectively (Teixeira et al., 2023; Helwig et al., 2023). The increasing demands on young football players, including frequent training sessions and competitive matches, place substantial strain on their bodies (Parr et al., 2021; Towlson et al., 2021).

Effective recovery strategies are essential to mitigate this strain and support the physiological adaptations that underpin performance improvements (Lee et al., 2023; Silva et al., 2022), which can help manage the physical and psychological stresses associated with intensive training and competition schedules (Teixeira et al., 2023; Howle et al., 2020). Optimizing recovery is crucial for youth players, whose bodies are still growing and developing, to support healthy development and avoid long-term health issues (Nobari et al., 2021; Clemente et al., 2021). Inadequate recovery and training intensity management during the microcycle can lead to overtraining syndrome, characterized by persistent fatigue, performance decline, and a heightened risk of injury (Ramos-Cano et al., 2022). Wearable technology has revolutionized the sports science field, providing insights into recovery states (Nobari et al., 2021; Clemente et al., 2021). Devices that monitor heart rate (HR)—a key indicator of autonomic nervous system function and recovery status—are now commonplace in youth sports settings (Teixeira et al., 2022a; Santos et al., 2021). Furthermore, wearable devices can track movement patterns and physical exertion using accelerometers and global positioning system (GPS) technology (Gómez-Carmona et al., 2021; Oliva-Lozano et al., 2020), providing detailed information on distances covered, speeds attained, and the intensity of movements during training and competition. Such comprehensive data collection offers a holistic view of an athlete’s workload and recovery needs (Oliva-Lozano et al., 2020).

The integration and analysis of this multifaceted data pose significant challenges, necessitating advanced analytical methods (Hessels et al., 2020). Machine learning (ML) has emerged as an artificial intelligence (AI) approach in this context, capable of analyzing vast and complex datasets to identify patterns and make predictions that traditional statistical methods might miss (Majumdar et al., 2022; Sarker, 2021). ML algorithms can process diverse data inputs, such as physiological demands and performance metrics, to classify and predict recovery outcomes (King et al., 2022; Filipas et al., 2020; Bourdon et al., 2017). This capability allows for a more sophisticated understanding of how different factors interact to influence recovery states, which is particularly significant in young athletes (King et al., 2022; Filipas et al., 2020; Bourdon et al., 2017). Recent studies highlight the effectiveness of ML models in predicting training load, recovery, and injury risks in football players (Vallance et al., 2023; Pillitteri et al., 2023; Rossi et al., 2022; Vallance et al., 2020). Vallance et al. (2023) demonstrated that tree-based models significantly improved perceived exertion predictions by 60%, with past RPE values being the strongest predictors. Pillitteri et al. (2023) demonstrated significant negative correlations between training load, recovery states, and model availability according to the training day. Rossi et al. (2022) emphasized the utility of the ML approach in predicting players’ wellness by integrating workload history, while Vallance et al. (2020) found that combining internal and external load features enhanced long-term injury risk prediction. All studies highlight the potential of ML for personalized training planning and injury prevention in football contexts (Vallance et al., 2023; Pillitteri et al., 2023; Rossi et al., 2022; Vallance et al., 2020).

However, ML is still being researched to manage recovery status in young sub-elite football players. Most studies focus on elite football players (Vallance et al., 2023; Oliver et al., 2020), leaving a critical need to investigate how training load and recovery variables manifest in different age groups and competitive levels (Teixeira et al., 2021a; Teixeira et al., 2022e). In addition, the application of ML models to classify recovery states in young footballers is still underexplored despite its potential to improve injury understanding and fatigue prediction (Teixeira et al., 2022e; Oliveira, 2023). This research has sought to address this gap by using training data to develop predictive models that optimize performance and wellbeing in sub-elite youth football players (Díaz-García et al., 2022; Coutinho et al., 2018). More specifically, this research aims to investigate the use of ML models in the classification of recovery states in sub-elite male football players in the U15, U17, and U19 age groups.

## 2 Methodology

### 2.1 Participants

A total of 20 U15 players (age: 13.2 ± 0.5 years; height: 1.69 ± 0.78 m; weight: 55.7 ± 9.4 kg), 20 U17 players (age: 15.4 ± 0.5 ± 1.2 y; height: 1.8 ± 0.5 m; weight: 64.38 ± 6.6 kg), and 20 U19 players (age: 17.39 ± 0.55 ± 1.8 ± 0.7 y; height: 1.82 ± 0.01 m; weight: 68.9 ± 8.4 kg) were observed for 2 weeks in a sub-elite Portuguese football academy. In the 2019–2020 competition season, the three age groups’ daily training loads were regularly observed. All participants were fully informed about the study’s purpose and potential risks in line with ethical standards. Informed consent was obtained from each participant or their guardian in the case of minors. The study protocol was approved by the local Ethics Committee at the University of Trás-os-Montes e Alto Douro (3379-5002PA67807).

### 2.2 Study design

The weekly training load was consistently monitored across three age groups during the first month of the 2019–2020 competitive season. The training data spanned a 6-week period, covering 18 training sessions and 324 observations (U15 = 41, U17 = 20, and U19 = 26 observations, respectively). Individual datasets were considered eligible if the player adhered to a one-game-per-week schedule and fully participated in the training sessions. The training cycle consisted of three weekly sessions, each lasting approximately 90 min, with match data excluded from the analysis. Training days were classified using the “match day minus format” (MD): MD-3 (Tuesday), MD-2 (Wednesday), and MD-1 (Friday). On average, each session involved 18 players. Each tier had week 1 (Week_1) and week 2 (Week_2) coded.

All age groups trained on outdoor pitches of official dimensions (FIFA standard; 100 × 70 m) with synthetic turf, held between 10:00 AM and 8:00 PM under similar environmental conditions (14–20°C; relative humidity 52–66%).

### 2.3 Procedures

Outfield players were tracked using portable GPS devices (STATSports Apex®, Northern Ireland) throughout each training session. The GPS units, sampling at 18 Hz, provided raw data on position, velocity, and distance and included an accelerometer (100 Hz), magnetometer (10 Hz), and gyroscope (100 Hz). Each player wore the micro-technology in a mini pocket of a custom-made vest provided by the manufacturer, positioned on the upper back between the scapulae. All devices were activated 30 min before data collection to ensure a clear satellite signal reception (Teixeira et al., 2021b; Beato et al., 2018). A 1-Hz short-range telemetry system was used to measure the heart rate (Garmin International, Inc., Olathe, KS, USA). The Rating of Perceived Exertion (RPE) scale was used to evaluate perceived exertion (Cabral et al., 2020). The total quality recovery (TQR) score proposed by Kenttä and Hassmén (1998) was applied to measure athletes’ recovery perception. The TQR was used before the start of the training session, while the RPE was applied after the end of the training session. The application steps were previously explained to the players, and a Microsoft Excel® spreadsheet was used to gather perceived exertion and recovery (Microsoft Corporation, USA) (Haddad et al., 2017).

### 2.4 Variables

The ML algorithms were built integrating age categories, anthropometric measures, GPS-based parameters, HR-based variables, and perceived exertion scales. Table 1 shows each included variable as well as the type of variable, the encoding label, and the average values.

**Table 1:** The variables included in the ML algorithm build.

Variable	Type of variable (Encoding or mean ± SD)
Age category (U-17, U-15 or U-19)	Binary numeric (positive = 1, negative = 0)
Height (meters)	Continuous numeric (1.73 ± 0.07)
Body weight (kg)	Continuous numeric (63 ± 10)
BMI (kg/m^2^)	Continuous numeric (20.6 ± 2.13)
Week (Week 1 or 2)	Binary numeric (positive = 1, negative = 0)
Position (CD, CM, FW, FB, WM)	Binary multiclass (combination of 0,1 sequences)
Total distance (meters)	Continuous numeric (5,317 ± 1,628)
rHSR (meters)	Continuous numeric (87 ± 78)
HMLD (meters)	Continuous numeric (560 ± 289)
AvS (repetitions)	Continuous numeric (51 ± 24)
SPR (repetitions)	Continuous numeric (7 ± 2)
DSL (repetitions)	Continuous numeric (252 ± 134)
ACC (repetitions)	Continuous numeric (46 ± 22)
DEC (repetitions)	Continuous numeric (42 ± 24)
Cal (kcal)	Continuous numeric (1,046 ± 354)
RPE (index)	Continuous numeric (13 ± 2)
TQR (index)	Continuous numeric (16 ± 2)
TQR_Class (recovery status)	Binary numeric (bad recovery = 1, good recovery = 0)

ACC, accelerations; AvS, average speed; Cal, Calories; CD: Central defenders; CM, central midfielders; DEC, decelerations; DSL, Dynamic stress load; FB, fullbacks; FW, forwards; HMLD, high metabolic load distance; rHSR, relative high-speed running; RPE, rating of perceived exertion; SPR, sprinting; TQR, total quality recovery; U, Under; WM, wide midfielders.

#### 2.4.1 Physical parameters

External training load was measured using time-motion data, including total distance (TD) covered (m), average speed (AvS), maximal running speed (MRS) (m/s), relative high-speed running (rHSR) distance (m), high metabolic load distance (HMLD) (m), sprinting (SPD) distance (m), dynamic stress load (DSL), number of accelerations (ACC), and number of decelerations (DEC). The GPS software provided data on locomotor categories above 19.8 km/h: rHSR (19.8–25.1 km/h) and SPD (>25.1 km/h). Sprints were tracked by number and average sprint distance (m). HMLD, a metabolic variable, represents the distance covered by a player when the metabolic power exceeds 25.5 W/kg. HMLD encompasses all high-speed running and accelerations and decelerations above 3 m/s^2^. Both acceleration variables (ACC/DEC) accounted for movements in the maximum intensity zone (>3 m/s^2^ and < 3 m/s^2^, respectively). DSL was assessed using a 100 Hz triaxial accelerometer integrated into the GPS devices, measuring the sum of accelerations across the three orthogonal axes of movement (*X*, *Y*, and *Z* planes), expressed as G force (Teixeira et al., 2021b; Beato et al., 2018).

#### 2.4.2 Heart rate

The HR and perceived exertion were applied to measure the recovery state. The maximum heart rate (HR_max_), average heart rate (AvHR), and percentage of HR_max_ (%HR_max_) were HR-based variables. HR_max_ was obtained by Yo–Yo Intermittent Recovery Test Level 1 (YYIR1) (Aquino et al., 2020). Training impulse (TRIMP) was obtained using the procedures suggested by Akubat et al. (2012). The TRIMP was calculated by multiplying training duration (min) intensity (ΔHR = AvHR – HR_rest_/HR_max_ – HR_rest_), which was weighted according to the fractional elevation in heart rate and blood lactate concentration (Akubat et al., 2012):


$$\mathrm{TRIMP}=\mathrm{training}\times \Delta HR\times 0.2053\;e^{3.5179\Delta HR}$$

#### 2.4.3 Perceived exertion

The RPE and TQR were obtained using a scale from 6 to 20 to assess players’ perceived effort and recovery states, respectively (Brink et al., 2010). A 2-week familiarization with both scales was conducted before the study. Data were collected individually by the same researcher during GPS device removal to prevent peer influence on recovery and effort perception (Kenttä and Hassmén, 1998; Haddad et al., 2017). A Microsoft Excel® spreadsheet (Microsoft Corporation, USA) was used to gather perceived data.

#### 2.4.4 Body composition

The height (m), weight (kg), chronological age (years), sitting height (cm), and level of experience (years) of the layers were recorded at each measurement point. Body mass index (BMI) was calculated by dividing weight by the square of height (kg/m^2^) (Teixeira et al., 2022a).

#### 2.4.5 Data preprocessing and normalization

We utilized the computational programming language PythonTM (Python, 2023), where the libraries “seaborn,” “matplotlib.pyplot,” “numpy,” and “pandas” were enabled to import, visualize, and conduct the necessary data transformations (Unpingco, 2016). The recovery state collected by the TQR score was targeted as a binary level (0 = well-recovered; 1 = insufficient recovery). Following the cutoffs suggested by Kenttä and Hassmén (1998), the positive label was considered with values <13 points in the TQR scale. To ensure that the classes would be well-defined and facilitate the decision boundaries characterization by the ML algorithms, we defined the negative value only for that player with scores equal to 19–20 in the TQR scale, or else, making that the points for insufficiently recovered and the well-recovered were far away from each other (More and Ingman, 2008). After applying this cutoff from the initial dataset (60 football players × 2 weeks = 120 observations), only 36 football players were included in the underlined criteria for positivity (*n* = 18 participants with TQR scores <13 points) or negativity (*n* = 18 participants with TQR scores approximately 19–20 points). To make possible the consideration of all features in calculating the importance, those features with a categoric nature were converted into numeric binary arrays using the one-hot encoding (Hancock and Khoshgoftaar, 2020). Next, the feature selection was performed using two different steps: the first step was performed where a correlation matrix was applied to identify the most correlated features and reduce dimensionality problems within the dataset, and in the second step, the random forest (RF) classifier was used to identify non-linear relationships between the most correlated features and thus build a more comprehensive panel of predictors of the football players’ recovery states. In the second step of the feature selection process, the “train_test_split” function was activated from the “sklearn” library, considering 70% of the dataset for training (*n* = 25) and 30% for testing (*n* = 11).

Furthermore, we employed the package “from sklearn.preprocessing import StandardScaler” to normalize the data after observing significant differences between the feature’s numerical scales and turned on the “StandardScaler” function (Unpingco, 2016; Biamonte et al., 2017). The characteristics were scaled within a range of −1,1 to facilitate easier interpretation of the sigmoid function as part of the normalizing process $\sigma \;\left(x\right)=\frac{1}{1+e^{-x}}$ [with binary data (0,1)], where “*e*” is the numerical basis of the classification algorithm and “*x*” is the independent variable (2.71828) (Narayan, 1997).

#### 2.4.6 Classifying algorithms

To perform the football players’ recovery state classification, we applied the rerun of the “train_test_split” function, also considering the same splitting setup [70% for training (*n* = 25); 30% for testing (*n* = 11)] (Unpingco, 2016; Cai et al., 2018). To guarantee reproducibility between various runs of the same code, we employed a random seed of 0 for all algorithms. Next, five ML classifiers were implemented using the libraries “sklearn.neighbors import KNeighborsClassifier” [(Rico-González et al., 2022b) for K-nearest neighbors classifier (KNN)], “from sklearn.ensemble import GradientBoostingClassifier” [(Kellmann et al., 2018) for Gradient Boosting Classifier (XGbosst)], “from sklearn.svm import SVC” [(Teixeira et al., 2022a) for support vector machine (SVM)], “from sklearn.ensemble import RandomForestClassifier” [(Teixeira et al., 2022b) for RF], and “from sklearn.tree import DecisionTreeClassifier” [(Teixeira et al., 2023) for DT Classifier] were activated to apply the algorithms and perform the recovery state classification (Python, 2023; Unpingco, 2016; Haslwanter, 2016; Pedregosa et al., 2011). Since all ML classifiers have limitations and strengths, the five ML classifiers were chosen in the present study aiming to verify the stability among different models to ensure that there were no overfitting and underfitting, thus testing their robustness to generalize to unseen datasets (Pedregosa et al., 2012; Kursa and Rudnicki, 2011).

The functions for accuracy, precision, recall, and F1-score were activated by activating the library “from sklearn.metrics import accuracy_score, confusion_matrix, classification_report” to assess the models (Hicks et al., 2022; Jierula et al., 2021). The following is a complete description of the algorithms and the corresponding assumptions:

#### 2.4.7 K-nearest neighbors classifier

A data point is classified by the KNN classifier in the feature space based on the majority class among its KNN (Uddin et al., 2022). The equation exemplifies KNN:


$$y=\mathrm{mode}\left(y_{\mathrm{neighbors}}\right)$$

where

- *y* is the predicted class label;
- *y*_neighbors_ is the class labels of the k-nearest neighbors; and
- ***mode*** is the most frequently occurring class label among the neighbors.

#### 2.4.8 Gradient boosting classifier

The XGBoost classifier is the algorithm that builds a sequence of trees in which the new tree corrects the errors of the previous trees by minimizing a loss function (Natekin and Knoll, 2013). This is the XGBoost equation expressed as follows:


$$F_{m}\left(x\right)=F_{m}-1\left(x\right)+\gamma_{m}h_{m}\left(x\right)$$

where

- *F_m_*(*x*) is the prediction of the *m*th model;
- *F_m_* − 1(*x*) is the prediction of the (*m* − 1)th model;
- *γ_m_* is the learning rate, which scales the contribution of each tree; and
- *h*_m_ is the *m*th weak learner (usually a DT).

#### 2.4.9 Support vector machine

SVM classifier locates the hyperplane in the feature space that most effectively divides the classes with the greatest margin (Cervantes et al., 2020). The SVM was expressed by


$$\mathrm{minimize}\;\left(\frac{1}{2}||w{\left|\right|}^{2}\right)\mathrm{subject to}\;yi\left(w.x_{i}+b\right)\geq 1$$

where

- *w* is the weight vector that defines the hyperplane;
- *b* is the bias term;
- *y_i_* is the class label of the *i*th training sample;
- *x_i_* is the feature vector of the *i*th training sample; and
- *w·x_i_ + b* is the decision function that calculates the distance from the hyperplane.

#### 2.4.10 Random forest classifier

The RF classifier builds several DTs and outputs the mode of the classes for classification (Breiman, 2001). The equation can be expressed by


$$y=\mathrm{model}\left(\left\{h_{t}\left(x\right)\right\}=rT1\right)$$

where

- *y* is the predicted class label;
- *h_t_* is the prediction from the *t*th DT;
- *T* is the total number of trees in the forest; and
- *mode* is the most frequently occurring class label among the trees’ predictions.

#### 2.4.11 Decision tree classifier

To maximize the separation of classes at each node, the DT classifier essentially operates by dividing the data into subgroups based on the most relevant feature (Song and Lu, 2015). DT is characterized by the following equation:


$$\mathrm{split criterion}:\mathrm{Gini}\left(t\right)=1-\overset{n}{\underset{i=1}{\sum }}p_{i}^{2}$$

where

- Gini(*t*) is the Gini impurity for a node *t*;
- *n* is the number of classes; and
- *p_i_* is the probability of a randomly chosen element being classified as class *i* at node *t*.

#### 2.4.12 Model evaluation

To assess the model’s performance, we used the metrics accuracy, precision, recall, and F1-score, as explained in the following (Hicks et al., 2022):

(1) Accuracy score: Accuracy measures the proportion of correctly classified instances among all instances. It is calculated as the ratio of correctly predicted instances (true positives and negatives) to the total number of instances (Hicks et al., 2022).

$$\mathrm{Accuracy}=\frac{TP+TN}{TP+TN+FP+FN}$$

where *TP* = true positives; *TN* = true negatives; *FP* = false positives; and *FN* = false negatives.

(2) Precision: Precision measures the proportion of predicted positive instances that are correctly classified. It is calculated as the ratio of true positives to the sum of true positives and false positives (Hicks et al., 2022).

$$\mathrm{Precision}=\frac{TP}{TP+FP}$$

(3) Recall: Sensitivity, also known as recall or true positive rate, measures the proportion of actual positive instances that the model correctly predicts. It is calculated as the ratio of true positives to the sum of true positives and false negatives (Hicks et al., 2022).

$$\mathrm{Recall}=\frac{TP}{TP+FN}$$

(4) F1-score: The F1-score is the harmonic mean of precision and recall, providing a single metric that balances both measures. It is calculated using the precision and recall values, combining them into a single value (Hicks et al., 2022).

$$F1-\mathrm{score}=2\times \frac{PPV\times \mathrm{Recall}}{PPV+\mathrm{Recall}}$$

To evaluate the models’ stability in the classification task, we employed K-fold cross-validation. This method divides the original dataset into K distinct subsets, where each subset is alternately used as a validation set while the remaining subsets are used for training. This approach assesses how consistently the models perform across different segments of the dataset, ensuring the robustness of the results (Wong, 2015). For this evaluation, we tested 5-fold of the original X array used in the training and testing processes of the five ML classifiers (Rodriguez et al., 2010). This approach allowed us to evaluate the consistency of the classifications.

## 3 Results

**Figure 1:**
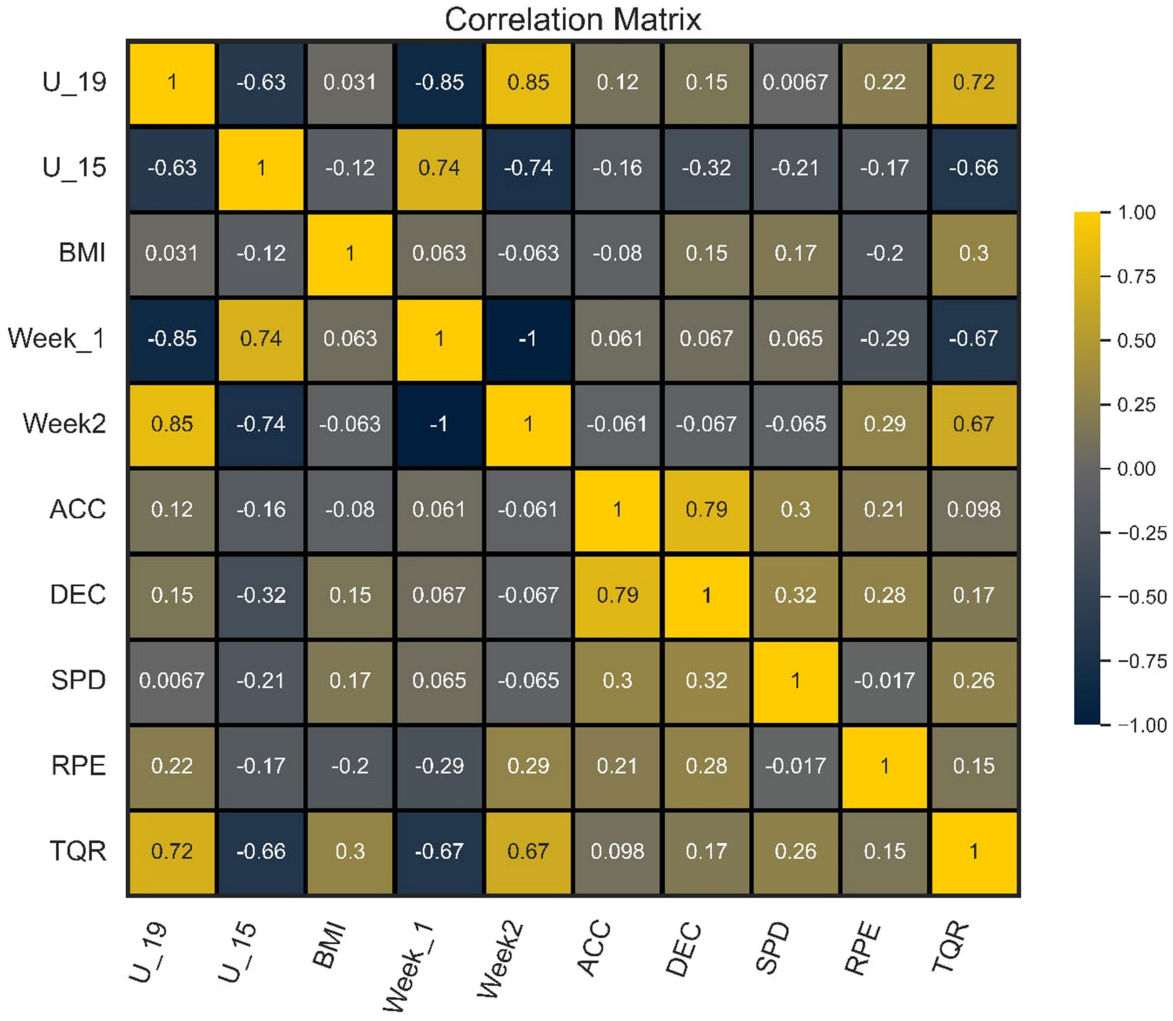
Correlation heatmap of features and TQR classes. ACC, accelerations; BMI, body mass index; DEC, decelerations; RPE, rating of perceived exertion; SPD, sprint distance; U_15, under 15; U_19, under 19; Week_1, first weekly training load; Week_2, second weekly training load.

Figure 1 shows the correlation coefficient of each independent variable with the TQR classes. In this way, we consider a panel consisting of only variables that presented at least small correlation coefficients with the target variable, fitting the dataset with the variables U19, U15, BMI, ACC, DEC, Week_1, Week_2, SPD, and RPE. These features were filtered within a new dataset, where they were considered for the final feature selection process with an RF classifier.

Next, the RF algorithm presents a very good classification report (accuracy = 92%; recall = 91%; and F1-score = 91%), with a good validation report after passing the same array within the 5-fold cross-validation (accuracy range = 71–87%; standard deviation = 12%; and average accuracy = 83%). Table 1 shows the classification report for the second step of feature selection with an RF classifier.

**Figure 2:**
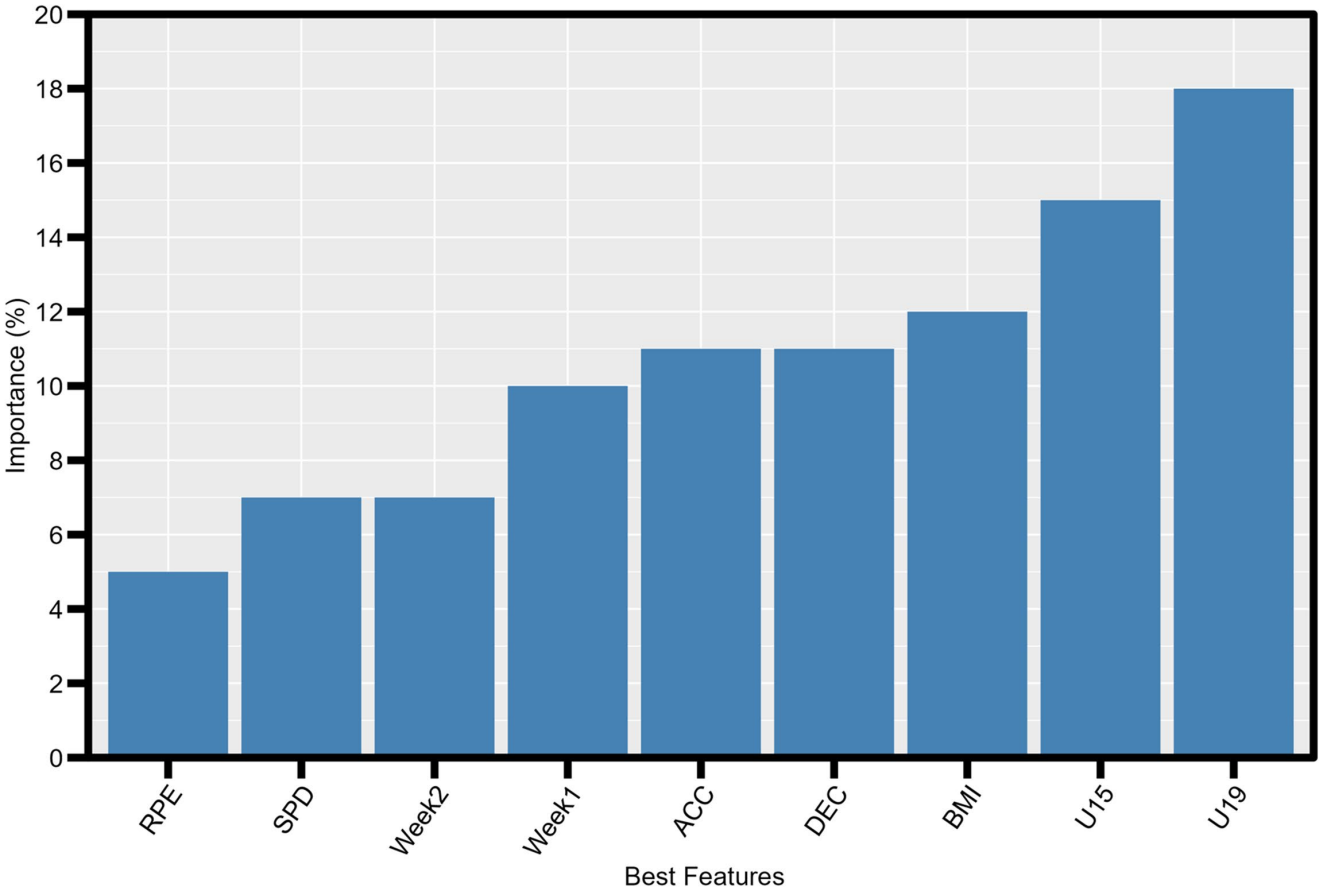
Best features to classify the soccer player’s recovery state. Data are displayed in percentage of importance. ACC, accelerations; BMI, body mass index; DEC, decelerations; RPE, rating of perceived exertion; SPD, sprint distance; U_15, under 15; U_19, under 19; Week_1, first weekly training load; Week_2, second weekly training load.

Figure 2 shows the best ranking of features captured by RF, reporting that the best features were U19 (18%) and U15 (15%) age categories, and the RPE (3%) presented the weaker contribution.

After reducing the data dimensionality, we implemented the five ML algorithms considering the panel of best features hierarchically reported as follows: U19, U15, BMI, ACC, DEC, Week_1, Week_2, SPD, and RPE. Table 2 shows that the algorithm’s performance ranged from 73–100% (Table 2).

**Table 2:** Detailed classification of random forest (RF) algorithm applied to feature selection.

	Precision	Recall	f1-score	Support
Class				
0	1.00	0.80	0.89	5
1	0.86	0.100	92	6
Metrics				
Accuracy			0.91	11
Macro avg	0.93	0.90	0.91	11
Weighted avg	0.92	0.91	0.91	11

Avg, average.

Table 3 compiled the cross-validation of the algorithm’s performance, which with an average performance of 79% validated and pointed to good generalization performance of the panel of features collected with wearable devices in predicting the football player’s recovery state (Table 4).

**Table 3:** Algorithm’s performance in classifying football’s fatigue states.

Algorithm	Accuracy (%)	Precision (%)	Recall (%)	F-1 score (%)	Average metrics
KNN	100	100	100	100	100
XGboost	73	74	73	73	73
SVM	100	100	100	100	100
RF	100	100	100	100	100
DT	100	100	100	100	100
Algorithm’s Aver.	95	95	95	95	95

KNN, k-Nearest Neighbors; XGBoost, Gradient Boosting Gradient Boosting Classifier; SVM, Support Vector Machine; RF, Random Forest Classifier; DT, Decision tree Classifier; Algorithm’s Aver, general algorithm’s average.

**Table 4:** Outputs of the cross-validation of the classifying models’ performance.

Algorithm	Accuracy (%)	Accuracy Sub.1 (%)	Accuracy Sub.2 (%)	Accuracy Sub.3 (%)	Accuracy Sub.4 (%)	Accuracy Sub.5 (%)	SD (%)
KNN	83	87	71	85	71	100	11
XGboost	75	75	57	71	71	100	14
SVM	75	62	71	71	71	100	13
RF	83	87	85	71	71	100	11
DT	78	75	71	57	85	100	14
Overall Performance (x̄)	79	77	71	71	74	100	13

Sub., subsets from the entire X array; SD, standard deviation; x̄., arithmetical average; KNN, k-Nearest Neighbors; XGBoost, Gradient Boosting Gradient Boosting Classifier; SVM, Support Vector Machine; RF, Random Forest Classifier; DT, Decision tree Classifier; Algorithm’s Aver, general algorithm’s average.

## 4 Discussion

The primary objective of this study was to investigate the use of ML models in the classification of male football players in the U15–17 and U19 age groups for recovery states. The key parameters offer a detailed picture of the physical and mental demands placed on players during training sessions. After reducing the data dimensionality, we implemented the ML algorithms considering a panel of 9 variables (U19, U15, BMI, ACC, DEC, Week_1, Week_2, SPD, and RPE). The 9 features were included according to their percentage of importance (3–18%). As the main results, we got good (73%) to very good (100%) in identifying football players’ recovery state based on the 10 feature panel football.

The correlation analysis revealed that several variables exhibited significant correlations with the target variable (TQR). These variables, including age categories, BMI, acceleration, deceleration, training weeks, speed, and both subjective and objective RPE, were selected for further analysis using the RF classifier. The RF algorithm demonstrated strong predictive performance, achieving an accuracy of 92% and an F1-score of 91%. Cross-validation further validated the model’s generalization ability, with an average accuracy of 83% across 5-fold. Feature importance analysis identified age categories as the most influential predictors, followed by RPE. Drawing from theoretical underpinnings and insights from existing studies in this area, the selected variables for the panel included SPR, HMLD, DSL, AvS, and ACC. These variables exhibited percentage importance ranging from 3 to 18%, signifying their significant relevance in predicting players’ recovery states. Implementing ML algorithms using this panel of five variables yielded varied performances. Both RF and DT algorithms demonstrated exceptional performance, each with an accuracy of 99%. This high performance can be attributed to the ability of these algorithms to effectively handle the complexity and non-linearity of the data, as well as their robustness to data variability. Furthermore, the insights from the existing literature focusing on applying ML in football contexts, training load monitoring, and related areas emphasize the importance of data-driven approaches and algorithm selection. Techniques such as RT and DT have been widely recognized for their effectiveness in sports analytics due to their ability to handle complex datasets and provide interpretable results. XGBoost, another algorithm utilized in this study, also exhibited high performance with an accuracy of 96%. This underscores its efficacy as a boosting technique that enhances predictive accuracy by combining multiple weak models into a robust model. In contrast, KNN and SVM algorithms demonstrated lower performances, with 51 and 40% accuracy, respectively. These findings suggest that KNN and SVM may not be as effective in dealing with the complexity of the training data collected via wearable devices. Recent advancements in sports science have significantly enhanced the analysis and monitoring of football players’ performance and wellbeing (Nobari et al., 2021; Clemente et al., 2021). Standard methods for analyzing player movement and fatigue, such as perceived exertion scales and heart rate monitors, have proven effective and accessible (Kenttä and Hassmén, 1998). These tools provide practical means for regularly assessing psychophysiological fatigue and performance changes during training and matches (Cabral et al., 2020).

The subsequent application of five ML algorithms to the selected features yielded consistent and promising results. All algorithms achieved accuracies ranging from 73 to 100%, with an average performance of 95%. The cross-validation confirmed the generalization performance of these models, demonstrating their ability to predict recovery states in football players based on the collected features. These findings suggest that a combination of age-related factors, physiological metrics, and subjective perceived assessments can effectively predict recovery states in young football players. This value reflects the weighted average accuracy of the different algorithms used in the study. While the individual top performances of RF and DT are noteworthy, the overall weighted average is influenced by the relatively lower performances of KNN and SVM algorithms. Therefore, practical applications should consider not only individual performance but also the robustness and consistency across different scenarios when selecting ML algorithms. ML models can achieve relatively high accuracy in predicting outcomes or analyzing data, and their performance can vary significantly depending on the specific algorithm used. In this study, the overall performance of the ML models, as indicated by a compiled algorithm performance table, was 74.5%, reflecting a weighted average accuracy. Therefore, when applying ML models in practical sports science scenarios, it is essential to consider not just the highest performing algorithms but also the robustness and consistency across various conditions and datasets (Unpingco, 2016; Cai et al., 2018). This comprehensive approach ensures that the chosen ML model performs reliably under different circumstances, enhancing its practical utility in sports science applications (Hicks et al., 2022; Jierula et al., 2021).

However, the study also highlights the variability in individual responses to training loads. The age group was a significant predictor of recovery status in a study that identified essential variables, including U19, U15, BMI, ACC, DEC, Week_1, Week_2, SPD, and RPE. Recent studies have demonstrated the effectiveness of these models in classifying young football players’ recovery states based on data collected from wearable devices (Majumdar et al., 2022; Rico-González et al., 2022a; Teixeira et al., 2024). This finding is consistent with the systematic study, highlighting the importance of integrating subjective wellness and training load indicators (Vallance et al., 2023; Herold et al., 2019). The RF classifier demonstrated these models’ reliability across various expertise levels, achieving an accuracy of 92% on the training set and maintaining an average accuracy of 83% in 5-fold cross-validation. This finding is consistent with a systematic review, highlighting the importance of integrating training load data with perceived wellness to improve predictive accuracy in football (Rico-González et al., 2022a). Majumdar et al. (2022) also observed that despite interpretability issues, black-box models such as RF often outperform other methods in predicting relationships between workload and injuries in football. Such insights are vital for developing customized training and recovery plans for individual athletes. Furthermore, feature importance analysis from the study highlighted the significant role of perceived exertion in recovery predictions to understand player development and injury prevention (Teixeira et al., 2024). The focus on subjective measures such as RPE and its link to objective training loads is further supported by research showing that wellness questionnaires can enhance monitoring in football (Calvo, 2019; García-Aliaga et al., 2021; Calvo et al., 2019). Moreover, testing different ML algorithms on a reduced feature set validated the effectiveness of the selected variables in predicting recovery states and fatigues with consistently strong accuracy (Calvo, 2019; Calvo et al., 2019). Calvo et al. (2019) recently reported that mental load influences recovery states, impacting decision-making, technical performance, and physical outputs. Changing the scoring structure during football practice has a substantial impact on the physical and mental strain of players; this effect is more pronounced in shorter games than in possession drills (Calvo, 2019). Fatigue can be effectively managed by modifying psychological content, task features, coaching behaviors, and competitive structure (Miguel et al., 2021; Oliveira et al., 2021). Further research should add variables to measure central and peripheral fatigue to compare them with recovery states and the possible value of perceived fatigability (Alba-Jiménez et al., 2022).

Despite a standardized training regimen, players exhibited different levels of perceived exertion and recovery (Teixeira et al., 2022a; Teixeira et al., 2022e). This variability underscores the need for individualized training plans that cater to the unique needs and capacities of each player. Coaches and sports scientists should consider these individual differences when designing training programs to optimize performance and reduce the risk of injury. Environmental conditions, such as temperature and humidity, were kept relatively consistent during the training sessions (Taylor et al., 2010). This controlled environment ensured that external factors did not unduly influence the training loads and recovery metrics. Nevertheless, the future studies could explore the impact of varying environmental conditions on training and recovery to provide more comprehensive guidelines for training under different climates. The findings from this study indicate that the training loads were systematically managed, with a clear structure to the training microcycle. The findings emphasize the importance of individualized training approaches and the need for ongoing monitoring to ensure the health and performance of young athletes (Howle et al., 2020). In addition, the results of this study provide valuable insights into the relative importance of independent variables in the dataset and their contribution to predicting the recovery state of football players using ML algorithms (Teixeira et al., 2023; Howle et al., 2020). This variable selection was crucial for reducing data dimensionality and facilitating the efficient implementation of ML algorithms. U19, U15, BMI, ACC, DEC, Week_1, Week_2, SPD, and RPE are crucial for predicting training demands in sub-elite young footballers.

### 4.1 Practical applications, the future research, and limitations

The future research should continue to explore the interplay between training load, recovery, and performance, incorporating a wider range of variables and more extended observation periods along the season. The integration of advanced monitoring technologies, such as GPS and accelerometers, has revolutionized the way training loads are assessed in sports (Hessels et al., 2020). These tools offer validated accuracy and granularity, allowing for more informed decision-making in training design and load management (Teixeira et al., 2021b; Teixeira et al., 2022d). The use of high-frequency sampling devices in this study ensured that even the subtle nuances of player movement and exertion were captured, providing a robust dataset for analysis. The RPE provided an additional layer of understanding by quantifying the subjective effort perceived by the players (Chang et al., 2020; De Meester et al., 2020). This measure is particularly useful for assessing internal load and ensuring that training intensities are aligned with the players’ physical capacities (Rico-González et al., 2022b; Sallen et al., 2020). The use of RPE has been validated in numerous studies and is recognized as a reliable indicator of training load in football (Teixeira et al., 2022c; Ferraz et al., 2022). All these variables are high-intensity variables, so monitoring them is essential to describe their impact to predict recovery states and prevent fatigue (Alba-Jiménez et al., 2022). This point plays a fundamental role in the application of complementary training methodologies associated with Strength and Conditioning, such as concurrent training (Seipp et al., 2023), plyometric (Gherghel et al., 2021), or strength, agility, and quickness (SAQ) (Trecroci et al., 2016; Trecroci et al., 2022). Moreover, the RPE session values could be another strategy for refining the recovery states classification model and to further individualize the training load. Another potential limitation, as the article currently stands, could be that a preliminary test was not conducted to determine the relationship between HR and lactate levels. This may have resulted in TRIMP not being a reliable predictor of recovery or fatigue. Thus, extending the monitoring periods over different seasons and including data from real match contexts may help to better understand long-term fatigue and recovery patterns. Thus, the future studies could incorporate other variables, such as biochemical markers, sleep patterns, and psychological measures, to enhance the predictive power of recovery models. The inclusion of biochemical data (stress and inflammation) and sleep patterns could also be very valuable for more profound comprehension of the recovery state during the weekly training process of football players during different sportive seasons (Branquinho et al., 2024a; Branquinho et al., 2024b).

In fact, using more advanced modeling, such as deep learning and time series approaches, could improve prediction accuracy. In addition, incorporating technical and tactical performance metrics alongside recovery data could provide more comprehensive insights into player readiness. The importance of age-related suggests that recovery management protocols should be tailored to specific age groups to ensure optimal recovery. The integration of GPS, HR data, and perceived exertion provides valuable insights that can be used to monitor recovery states during the season. Furthermore, these enhancements could further refine models and algorithms for recovery protocols and injury prevention strategies in youth football.

As research limitations, data were collected from the unreal context of football matches. There is a lack of longitudinal data that would help to understand long-term patterns of fatigue and recovery state among football players. In addition, the predictor explained between 3 and 18% of recovery status, suggesting that additional predictors could improve the accuracy of the model. In fact, the low training frequency per week (3 days vs. 4 days without activity) makes it essential to monitor other activities outside the training period to understand the influence of fatigue and the ability of the models studied to explain recovery. Thus, additional longitudinal data are essential in training algorithms that are more representative of young football players. More specifically, we need to understand the effects of recovery states on other vital dimensions, such as technical and tactical performance at different levels, ages, and development stages (De Meester et al., 2020; Branquinho et al., 2024a).

## 5 Conclusion

In conclusion, the five ML models, in combination with weekly data, demonstrated the efficacy of wearable device-collected features as an efficient combination in predicting sub-elite young football players’ recovery states. Critical variables were identified by feature selection, and 10 variables—body mass, U15, U19, accelerations, decelerations, training weeks, sprint distance, and RPE—were taken into consideration while implementing the machine learning algorithms. The future research could explore incorporating technical, tactical, and psychological variables and applying deep learning techniques to potentially further improve the predictive accuracy and practical utility of ML models in the team’s sports contexts.

## Data Availability

The raw data supporting the conclusions of this article will be made available by the authors, without undue reservation.
